# Information-Theoretical Analysis of the Neural Code in the Rodent Temporal Lobe

**DOI:** 10.3390/e20080571

**Published:** 2018-08-03

**Authors:** Melisa B. Maidana Capitán, Emilio Kropff, Inés Samengo

**Affiliations:** 1 Departament of Medical Physics, Centro Atómico Bariloche and Instituto Balseiro, Comisión Nacional de Energía Atómica, Consejo Nacional de Investigaciones Científicas y Técnicas, 8400 San Carlos de Bariloche, Argentina; 2 Fundación Instituto Leloir, Consejo Nacional de Investigaciones Científicas y Técnicas, 1425 Buenos Aires, Argentina

**Keywords:** mutual information, synergy, redundancy, neural code, hippocampus, entorhinal cortex, navigation

## Abstract

In the study of the neural code, information-theoretical methods have the advantage of making no assumptions about the probabilistic mapping between stimuli and responses. In the sensory domain, several methods have been developed to quantify the amount of information encoded in neural activity, without necessarily identifying the specific stimulus or response features that instantiate the code. As a proof of concept, here we extend those methods to the encoding of kinematic information in a navigating rodent. We estimate the information encoded in two well-characterized codes, mediated by the firing rate of neurons, and by the phase-of-firing with respect to the theta-filtered local field potential. In addition, we also consider a novel code, mediated by the delta-filtered local field potential. We find that all three codes transmit significant amounts of kinematic information, and informative neurons tend to employ a combination of codes. Cells tend to encode conjunctions of kinematic features, so that most of the informative neurons fall outside the traditional cell types employed to classify spatially-selective units. We conclude that a broad perspective on the candidate stimulus and response features expands the repertoire of strategies with which kinematic information is encoded.

## 1. Introduction

Hippocampal and entorhinal neurons are selective to the kinematic state of the subject [1]. In the original studies, selectivity was assessed by constructing firing maps of individual neurons, that is, by describing the dependence of the firing rate as a function of the position of the animal [2,3]. These representations can be used to estimate the amount of encoded spatial information [4]. Firing maps constitute the two-dimensional generalizations of the tuning curves employed in sensory domains [5]. In addition to neurons selective to position, other cells responding to head direction [6], velocity [7], environment boundaries [8], direction of motion [9], and combinations of these features [10], have been found. Moreover, not only the firing rate, but also the phase-of-firing with respect to the oscillatory extracellular local field potential (LFP) encodes kinematic information [11,12].

Tuning curves reveal how the mean firing rate, averaged inside a certain window and also across trials, is modulated by the stimulus. However, the neural code may well depend on other statistical properties of the joint distribution between stimuli and responses, beyond the mean [13]. If the variance of the firing rate were also modulated by position, for example, tuning curves would not be capturing part of the information encoded in responses. Shannon’s mutual information, defined as the Kullback-Leibler divergence between the joint distribution between stimuli and responses and the product of the marginals, is the most general tool to address all possible encoding mechanisms, since it makes no assumptions about the nature of the probabilistic mapping [14].

A delicate matter, however, is how to choose the set of stimuli and the set of responses between which information is to be calculated. In the sensory domain, this choice has been typically approached with two different strategies. The classical strategy has been to select a certain number of static or stereotyped stimuli using heuristic criteria, and to present them repeatedly to a subject while recording the neural responses in a given brain area. To apply this strategy, scientists need to know from the start the stimuli that are adequate to probe the area under study. For example, when examining early visual areas, gratings of varying contrast, orientation and spatial frequency are usually considered adequate; in the infero-temporal cortex, faces, hands, or tools are often employed; in the temporal lobe of navigating rodents, the position, head direction or direction of motion of the animal are taken as stimuli. Of course, it may well be the case that, by probing a given brain area with the wrong stimuli, one misses entirely the key carriers of information. In fact, the identification of the relevant stimuli has often contained a certain dose of luck, as for example, the discovery of orientation selectivity in V1 [15], of hand or face selective neurons in IT [16], and place cells in CA1 [17]. One may therefore wonder whether still more appropriate stimuli could be found, better than the ones traditionally employed, if only more stimuli were tested.

The second strategy circumvents the arbitrariness in the selection of stimuli by no longer considering them isolated items. The stimulus is seen as a continuous signal that flows into the area under study, as happens in ecological conditions. The underlying assumption is that, if the ensemble from which stimulus are drawn is similar to the natural environment, eventually, the relevant features will come up [18,19]. This approach has allowed scientists to discover many relevant stimulus features that had not been noticed using the classical strategy, as for example, multiple subfields in macaque V1 [20], or the correlated deflection of several whiskers in barrel cortex [21]. The information-theoretical formulation of this strategy was provided by Strong et al. in 1998 [22]. Stimuli were considered to be stochastic processes unfolding in time, and the code was characterized by the information rate, that is, the mutual information per unit time between a set of responses and the set of all stimulus histories contained in the input signal. From the operational point of view, each point in time can be associated with a specific stimulus history. The mutual information between stimuli and responses, hence, quantifies the difference between the response distributions conditional to a given point in time, and the response distributions marginalized over time. To calculate this information, the animal must be exposed to exactly the same stimulus sequence in repeated trials. The appeal of this method is that it makes no assumptions about the stimulus feature the response is selective to, nor whether selectivity is encoded in the mean or the variance of the response distribution. The drawback, is that selectivity is quantified, but the key informative features remain unknown.

In an attempt to develop data analysis techniques that are as open minded as possible with respect to the candidate stimulus features that may be relevant, as well as the candidate response features instantiating the code, here we adapt this strategy to the specific case of the encoding of kinematic variables in the temporal lobe. This is a challenging system, because exposing the animal to a rich set of stimuli requires its active engagement in locomotion, exploring different places and states of motion with a repetitive protocol. To this aim, we analyzed physiological data recorded in animals that in repeated trials followed exactly the same trajectory, as fixated by a careful experimental design. The paradigm allowed us to compare the response probability distributions conditional to one point along the trajectory, with the marginal distribution. We thereby assessed whether specific points in time produced distinctive responses, irrespective of whether such points could be linked to well-known kinematic features as “position”, “velocity” or “direction of motion”. The information thus obtained was then compared to the mutual information between traditional kinematic features and neural responses. We find that, in agreement with another previous open-minded approach developed with different data analysis techniques [23,24], traditional descriptions of the neural code capture only a limited fraction of the whole repertoire of encoding strategies.

To broaden also the candidate response features encoding information, we not only analyzed the traditional information-carriers, as firing rate and phase-of-firing with respect to the theta-filtered LFP, but also the delta-filtered LFP, since both the hippocampus and the entorhinal cortex contain a significant amount of power in the delta band. We also estimated the degree up to which the three candidate codes (rate, theta and delta) tend to coincide in the same cells, or are rather segregated into different populations. In addition, we also estimated the degree of synergy or redundancy between pairs of kinematic features, or pairs of response variables. We conclude that, by broadening both the stimulus and responses variables that can putatively encode information, the neural code in the temporal lobe represents kinematic information employing a wider collection of attributes than revealed by traditional approaches.

## 2. Results

A Long Evans rat was trained to run along a 4m linear track, inside a bottomless cart, the position and velocity of which was controlled by a computer (Section 4.1 contains the details of the behavioral task). Multiple trials were thus recorded, with identical kinematic conditions. In each trial, the rat ran first to the right, with two different velocities, while the activity of individual neurons in the hippocampus (H) and the entorhinal cortex (EC) was recorded, together with the LFP. When the animal reached the end of the track, it turned around, and the cart moved leftward returning to the starting point, again employing two different velocities. In this paper, several stimulus attributes are probed. The most general attribute is “Time along the trajectory”, henceforth shortened as “Time”. This attribute has the advantage of tagging whatever feature (among those that are repeated across trials) that may modulate the response. Relevant stimulus features may be traditional *kinematic variables*, as “Position”, or “Velocity”, but they may also be some event so far unnoticed by us scientists, as for example, a certain distress produced by the cable attached to the head of the animal when a specific motor action is required, or the awareness that the arrival to the end of the track is imminent, where the food reward is delivered. To determine the degree up to which the information encoded in the variable Time corresponded to the traditional kinematic variables, we introduced the variables Position, Velocity, Direction, SignedPosition and SignedDirection by adequately grouping temporal bins (Section 4.2 describes how different variables were defined and binned).

### 2.1. Population Firing Statistics

**Figure 1:**
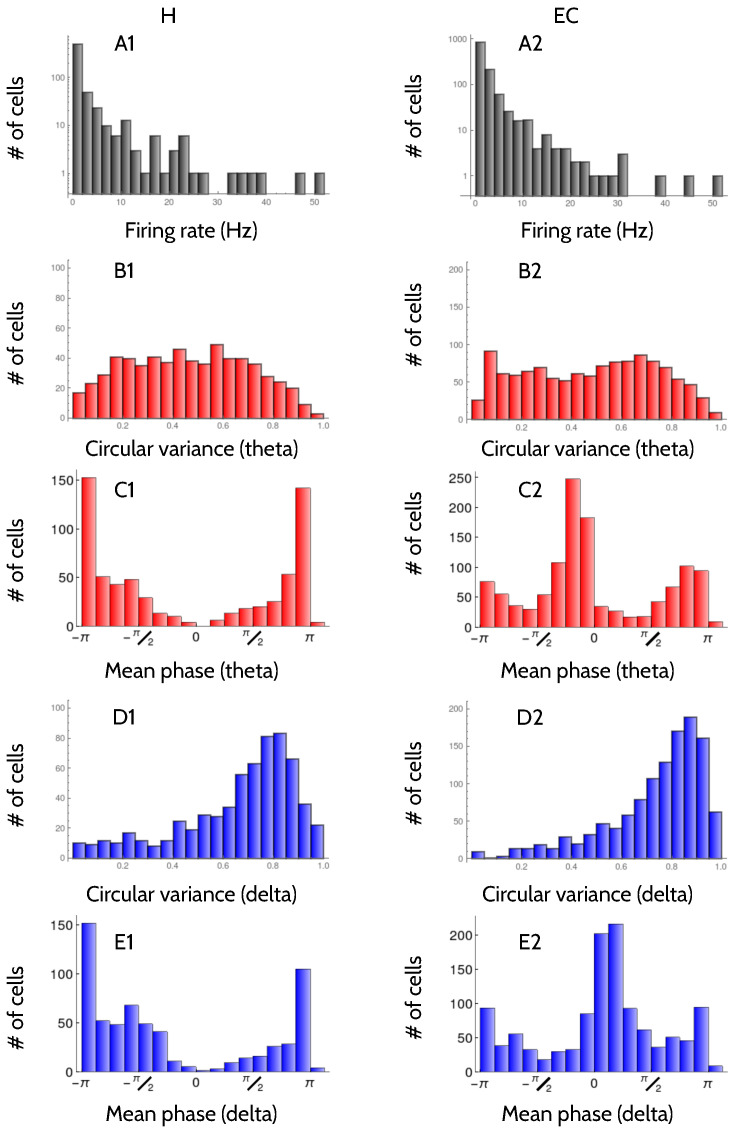
**Distribution of response properties in the recorded neurons**. Left column: Hippocampus. Right column: Entorhinal cortex. A: Histograms of the firing rate across the population. Vertical axis in logarithmic scale. B: Histograms of the circular variance of the phase-of-firing with respect to the theta-filtered LFP. C: Histograms of the mean phase of firing in the theta-filtered LFP. D: Same as B for the delta-filtered LFP. D: Same as C for the delta-filtered LFP. All the circular variances reported here are significant (Section 4.4).

In Figure 1, the distribution of firing rates and circular variances throughout the population of recorded neurons are displayed. Equations (1) and (2) in Section 4.4 define the relevant circular variances, as well as the criteria to assess their significance. The distribution of hippocampal (left) and entorhinal (right) firing rates is roughly exponential for low-to-medium frequencies, with a long high-frequency tail extending to the right. In the hippocampus, the mean firing rate is 2.2 ± 5.5 Hz, and in the entorhinal cortex, 2.3 ± 4.1 Hz. The two mean values are not significantly different (Student *t*-Test p=0.9), but the shapes of the distributions are significantly different (Smirnov-Kolomogorov p<0.0001), mainly due to the difference in the two variances. The average firing rate in the two regions is therefore similar, but in the H there is a larger cell-to-cell dispersion.

Section 4.3 describes the way the LFP is filtered in either the theta or the delta bands. The distribution of circular variances of the phase-of-firing with respect to the theta-filtered LFP is fairly broad, in both areas, with some cells with values even below 0.1 (Figure 1(B1,B2)). In H, the mean circular variance was 0.46 ± 0.24, and in the EC, 0.48 ± 0.26 Hz. The two mean values are not significantly different (Student *t*-test p=0.2). The shape of the distributions may or may not be considered significantly different, depending on the chosen significance threshold (Smirnov-Kolmogorov p=0.02). Hence, the amount of locking to the theta rhythm in both regions is comparable. In H, cells locked to theta preferred to fire at phase π (Figure 1(C1)), that is, when the LFP reaches a minimum. Instead, in EC the distribution of locking angles was bimodal, with peaks near 0 and π, and a preference for the former (Figure 1(C2)).

Neurons are less locked to the delta-filtered LFP than to theta. For delta, the distribution of circular variances peaks at around 0.8 in H, and in 0.9 in the EC, and with means 0.66 ± 0.23 and 0.73 ± 0.20, respectively (Figure 1(D1,D2)). The two distributions are also narrower than in the case of theta. The distributions obtained in H and EC are significantly different (Smirnov-Kolmogorov p<0.0001), and so are the two mean values (Student *t*-test p<0.0001). Hippocampal cells are more locked to the delta rhythm than entorhinal cells. With respect to delta, hippocampal cells preferred to lock at phase π (Figure 1(E1)), whereas entorhinal cells were divided into two populations, one of them locked at around phase 0, and the other at phase π, the former more numerous (Figure 1(E2)).

Cells that are tightly locked to a rhythm cannot transmit information using a phase code, because the total entropy is small. Cells whose phase-of-firing varies substantially, and the variation is not systematically related to kinematic variables, have no information either, because the noise entropy is large. Hence, neurons can only encode information in the phase of firing if they are locked to a specific phase, and the locking varies with some kinematic condition, either by shifting the preferred firing phase (the mean phase), or the degree of locking (the circular variance).

### 2.2. Example Neurons with Different Encoding Strategies

**Figure 2:**
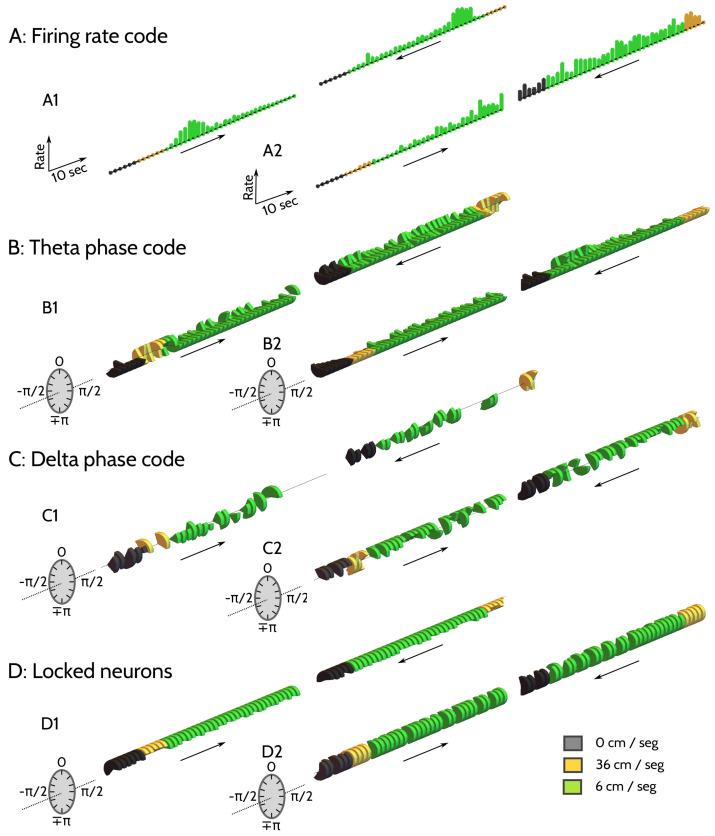
**Example codes**. Mean firing rate (**A1**,**A2**), and phase-of-firing with respect to the theta- (**B1**,**B2**,**D1**), and delta-filtered (**C1**,**C2**,**D2**) LFP. Horizontal axis is time, flowing from left to right. Right and left runs are displayed separately. Arrows indicate direction of motion. (**A**) Maximal rates: 130 Hz (**A1**) and 95 Hz (**A2**); (**B**–**D**) Mean and SD of phases-of-firing represented disc sectors covering $\langle \phi \rangle \pm \sqrt{-2\ln(\sigma_{\phi }^{2})}$ (Equations (1) and (2)). H: (**A2**,**B1**,**B2**,**C2**,**D1**). EC: (**A1**,**C1**,**D2**).

A visual inspection of the response properties of individual neurons revealed the existence of firing rate codes (Figure 2A) and phase-of-firing codes, the latter defined in terms of both the theta (Figure 2B) and delta phase (Figure 2C) of the LFP.

The entorhinal neuron of Figure 2(A1) shows selectivity to position. Firing rate is maximal when the rat is located in a region around 220 and 250 cm away from the starting point. The hippocampal neuron of Figure 2(A2) is instead selective to direction of motion. The firing rate is maximal when the rat is running towards the left. The hippocampal neuron of Figure 2(B1) encodes velocity with the mean phase-of-firing with respect to the theta-filtered LFP. The phase is almost always locked at π, except for high velocity, where it shifts to −π/2. In the hippocampal example of Figure 2(B2), the circular variance is fairly constant throughout the trial, except for the initial interval during slow running in the return journey, where it increases. This is an example of information encoded in a modulation of the response variance. In the entorhinal neuron of Figure 2(C1), the delta-filtered firing phases are mostly locked to π/2, although they occasionally shift towards the angle 0, most notably, at high velocities, and towards the end of the track during the rightward journey. The delta phases of the hippocampal example of Figure 2(C2) are mostly locked to 0 degrees, except at high velocities, and at a spot located approximately at 1 m from the end of the track . The firing phases of the two neurons of Figure 2D (hippocampal in D1 and entorhinal in D2) remain locked all throughout the trajectory (delta and theta bands, respectively).

### 2.3. The Encoding of the Variable Time by Individual Neurons

**Figure 3:**
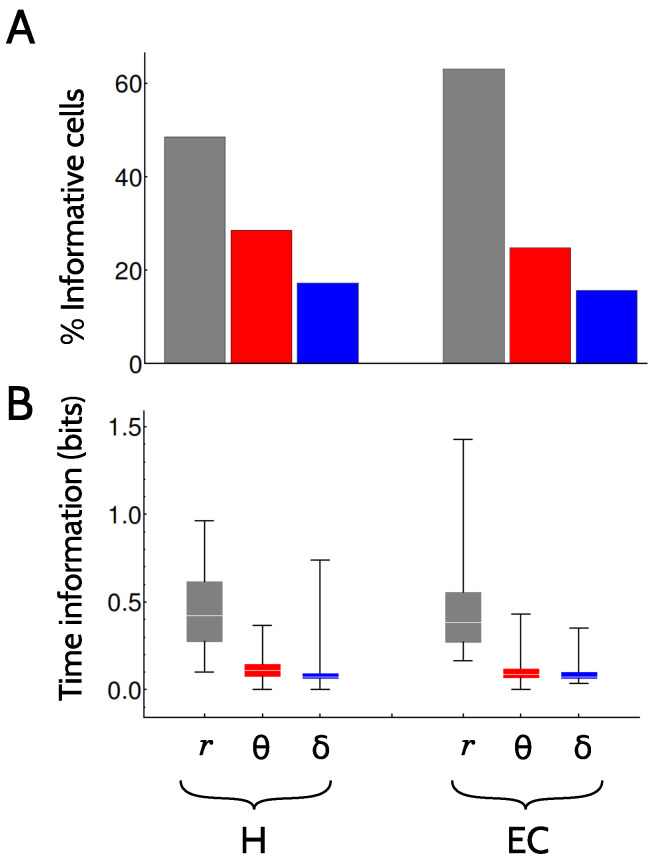
**The encoding of the variable Time by individual neurons**. (**A**) Percentage of cells that were informative about Time in the two brain areas (H and EC) and the three tested codes (firing, theta and delta). (**B**) Box-histograms of the mutual information I(Y;Time) of the cells with significant amounts of information for different choices of the physiological variable *Y*. The horizontal line represents the median, boxes extend between percentiles 25 and 75, and whiskers reach out from percentile 5 to 95.

In the behavioral task explored here, position, velocity and direction of motion are all functions of Time. Hence, the data processing theorem ensures that the mutual information between a physiological variable (as firing rate, or the phase with respect to the theta, or the delta-filtered LFP) and Time is at least as large as the information about any other derived kinematic feature. Section 4.5 describes the procedure we used to calculate all mutual informations. Figure 3 displays the dependence of the information about Time on each code.

In both brain areas, the most informative response feature was the firing rate, both regarding the number of cells encoding significant amounts of information (Figure 3A), and the actual amount of information (Figure 3B). Approximately half (49%) of the hippocampal cells transmitted Time information in their firing rates, and 63% cells in the entorhinal cortex. The difference between these two percentages is significant (Section 4.5 describes the significance criteria). The information encoded in the theta and delta phases, although substantially lower, is not negligible. The fraction of cells encoding significant amounts of information in the theta phase is 29% in H and 25% in EC, and the difference is significant. Fewer cells are involved in the delta code, 17% in H and 16% in EC.

Although the mean information transmitted with the two phase codes is noticeably lower than the mean of the firing rate code (about a quarter), there are a few neurons in the population with high information values (see the tails of the distributions in Figure 3B, indicated by the whiskers extending up to the percentile 95). Table A1 in Appendix B summarizes the mean, standard deviation and maximal information values of Figure 3B. A few outlier cells, hence, encode amounts of information in their firing phases that are comparable to the average information in the rate code.

**Figure 4:**
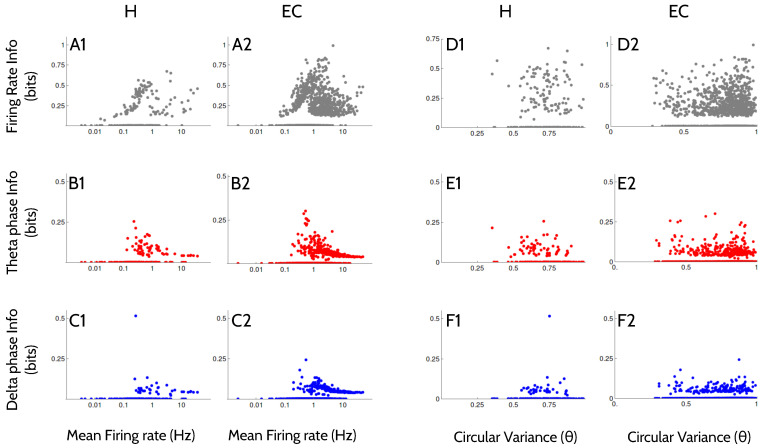
**Relation between firing properties and information about Time**. In each panel, each data point is a different cell. Cells that encoded an amount of information that was not significant appear clustered on the horizontal axis (the information value was set zero). Pearson correlation coefficients between the values displayed in the horizontal and vertical axes (including only the cells with significant amounts of information) are 0.04 (**A1**); −0.11 ${}^{*}$ (**A2**); −0.35 ${}^{*}$ (**B1**); −0.37 ${}^{*}$ (**B2**); −0.16 ${}^{*}$ (**C1**); −0.42 ${}^{*}$ (**C2**); where the asterisk indicates that the value is significantly different from zero at the 0.01 level. All correlation values in panels (**D**–**F**) are non-significant.

We then characterized the firing properties of the neurons that encoded significant amounts of information about the variable Time (Figure 4).

The cells with the lowest mean firing rates encoded amounts of information that were not significant, with all three codes (Figure 4A–C). As the mean firing rate increased beyond 0.1 Hz, the information encoded in the rate code increased linearly with the firing rate, until firing rates of approximately 1 Hz, where the information dropped again (Figure 4A). Instead, the information encoded in the phase-of-firing with respect to the theta and the delta-filtered LFP was a monotonically decreasing function of the mean firing rate (Figure 4B,C).

The degree of locking to the theta rhythm was not related to the information encoded by any of the three codes (Figure 4D–F). Similar results are obtained when the degree of locking is calculated with respect to the delta-filtered LFP (not shown).

An important question regarding the strategy of population signaling is whether the different neural codes are independent from each other. Independent codes would mean that the probability that a neuron encode significant amounts of information with one code (for example, the rate code) does not affect the probability that it employ another code (theta or delta). If codes are not independent, there are two possibilities. On the one hand, neurons may tend to come in disjoint categories, each category specialized in one specific code, for example, rate neurons, theta neurons, or delta neurons. This alternative would be expected if one of the codes, for example, the rate code, required certain biophysical properties that were not compatible with some other code, say, the delta or theta codes. The opposite situation would mean that neurons would tend to be either informative or not informative, and within the informative set, the probability for two or more neural codes to coexist should be higher that in the independent case. This alternative would be expected if the properties that make a neuron suitable for one code could also be exploited by another code.

**Figure 5:**
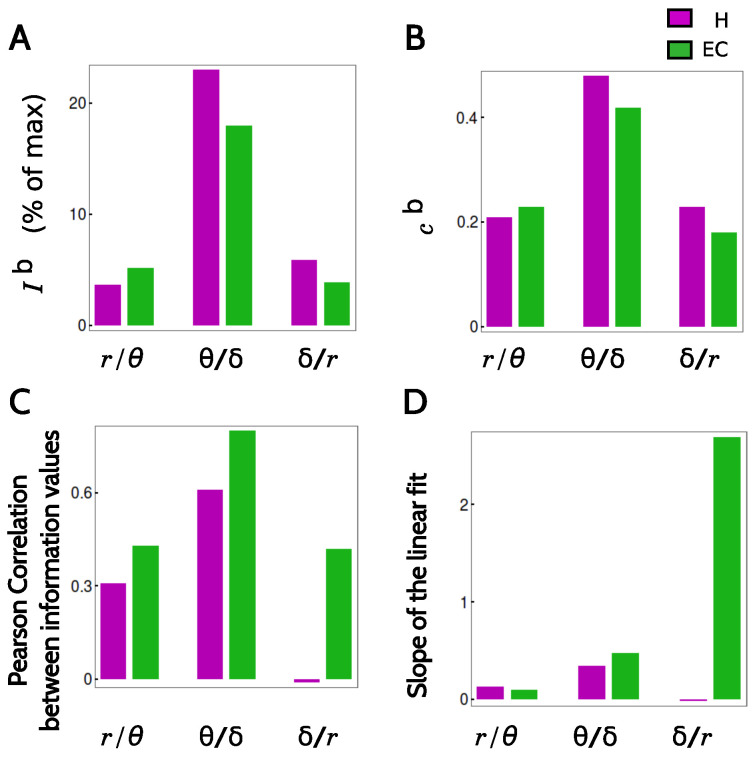
**Degree of overlap between pairs of neural codes**. (**A**) Mutual information $I^{b}\left(C_{i};C_{j}\right)$ defined in Equation (7). Information values are relative to the maximal possible information, (see Table A3 for the values in bits); (**B**) Pearson correlation coefficients $c_{ij}^{b}$ defined in Equation (8); (**C**) Pearson correlation between the mutual information I(i;Time) and I(j;Time) with *i* and *j* in {firingrate,θphase,δphase}; (**D**) Slope of the linear fit linking the information values of each pair of codes. In (**C**,**D**) only neurons encoding significant amounts of information with both tested codes are considered.

To assess the independence between codes, we verified if the knowledge that a given cell did or did not encode a significant amount of information with one code could be used to predict whether it also used another code. To this end, we defined the binary variables $C_{r},C_{\theta },C_{\delta }$ representing whether a given cell did or did not encode significant information about the variable Time with the codes rate, theta and delta, respectively. We then estimated from the data the distributions $P_{ij}\left(c_{i},c_{j}\right)$, with i,j belonging to {r,θ,δ}, and evaluated whether they coincided or not with the independent hypothesis, which predicts a joint distribution equal to the product of the marginals $P_{i}\left(c_{i}\right)\;P_{j}\left(c_{j}\right)$. The evaluation was performed in terms of the mutual information $I^{b}\left(C_{i};C_{j}\right)$ (Section 4.8 displays the mathematical definition of $I^{b}$). In both brain areas, and with all three pairs of neural codes, $I^{b}$ was found to be positive (Figure 5A), implying that the rate, theta and delta codes were not independent. To determine whether pairs of codes tended to coexist or, on the contrary, tended to be segregated into disjoint populations, we calculated the Pearson correlation coefficient $c^{b}\left(c_{i},c_{j}\right)$ (mathematical definition in Section 4.8), obtaining always positive values (Figure 5B). The rate code was only marginally (though significantly) correlated with each of the phase codes. The joint occurrence of the theta and delta codes was more marked, with correlation coefficients of 0.5 in H and 0.4 in EC.

Having determined that the populations of cells using one code or the other have a tendency to coincide, we assessed whether the amounts of encoded information was also correlated (see details in Section 4.8). This second analysis involved real (not binary) variables, representing the pairs of values of mutual information about the variable Time transmitted with any two codes. We only included the cells that transmitted significant amounts of information with the two tested codes (both binary variables equal to 1). The result is displayed in Figure 5C. Except for the comparison between delta and rate codes in hippocampus, all Pearson correlation coefficients were significantly positive. The information values that were most strongly correlated were those involving the theta and delta codes, with Pearson coefficients of 0.6 in H, and almost 0.8 in EC. In both areas, the correlation of the rate information values and any of the phase codes was lower.

In Figure 5D, the slope of a linear fit between the information encoded by each pair of codes is displayed. The data points to be fitted are ordered as pairs (x,y) with components (Inforate,Infotheta), (Infortheta,Infodelta), (Infordelta,Inforate). The slopes obtained in both areas for the pairs (rate, theta) and (theta, delta) are smaller than unity, which is consistent with the fact that the information values encoded by each individual neuron are typically ordered as Info(rate) > Info(theta) > Info(delta). The large value obtained for the pair (delta, rate) is also consistent with this ordering, since by transitivity, Info(delta) < Info(rate).

### 2.4. The Encoding of other Kinematic Variables by Single Neurons

**Figure 6:**
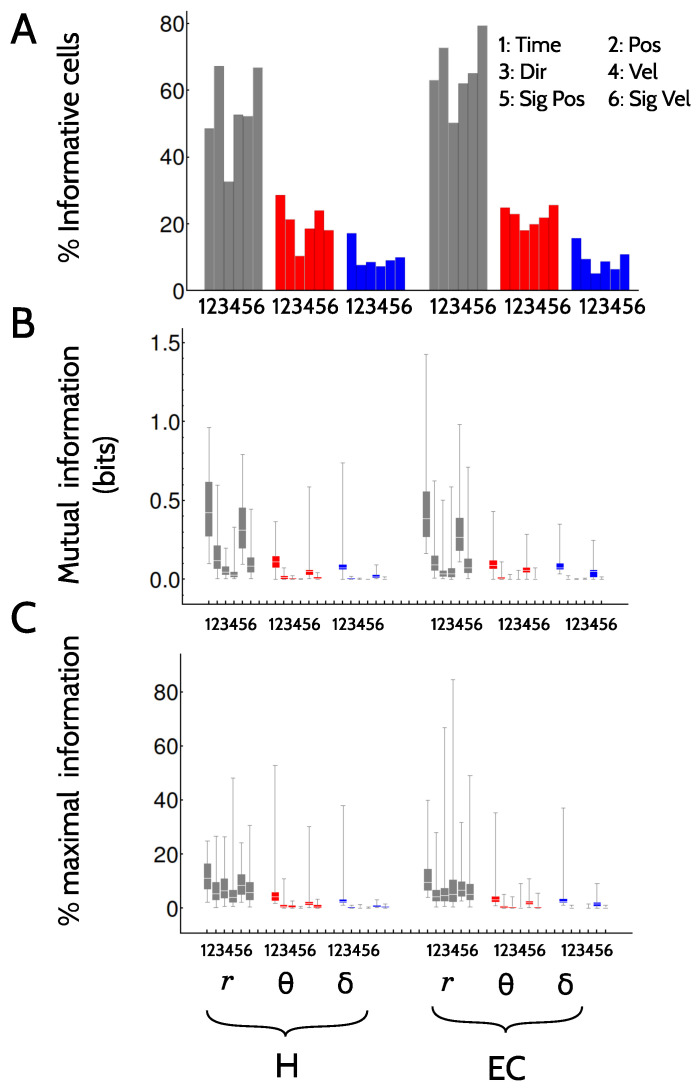
**Encoding of multiple kinematic variables by single cells**. (**A**) Percentage of cells that were informative about the different kinematic features in the two brain areas (H and EC) and the three tested codes (firing, theta and delta); (**B**) Box-histograms of the mutual information I(Y;Feature) for different choices of the physiological variable *Y* and the kinematic feature. Only cells that passed the significance test of Section 4.5 are included; (**C**) Same as (**B**), but with information values normalized by the maximal attainable information (the entropy of the set of kinematic features). Table A2 in the Appendix summarizes the mean, standard deviation and maximal information values of panel (**C**). Parameters of box histograms: same as in Figure 3.

Figure 6 describes the encoding of other kinematic features (Position, Direction, etc.).

In all tested kinematic features, just as it happened with the time variable, the firing rate code was the one that recruited the largest numbers of cells, typically duplicating the number involved in the delta and theta codes (Figure 6A). The latter, however, still represented between 15 and 25% of the population. In the rate code, the kinematic features that recruited the largest number of cells were Position and SignedPosition, surpassing even the number of cells that encoded Time. This result derives from the fact that the larger number of bins of the variable Time make its significance analysis stricter than with the other features. In all three codes, Direction was the kinematic feature that recruited the smallest numbers of neurons. In the delta phase code, there was a noticeable difference between the number of cells recruited by the Time variable, and all other kinematic features.

Figure 6B displays the raw information values, so as to determine whether the information encoded in the variable Time can or cannot be interpreted in terms of one of the classical kinematic features. In the firing rate code, the information encoded in the variable Time is explained to a large extent by SignedPositon, in both areas. In the two phase codes, instead, the information in SignedPositon represents a smaller fraction than the information in Time. This result, combined with the fact that Time recruits significantly more cells (panel A), suggests that phase codes (and specifically, the delta code) represent specific conjunctions of position, velocity and direction, that cannot be described by a single tested feature. Indeed, such conjunctions are evident in the examples of Figure 2(C1,C2).

In Figure 6C, the information is normalized with the entropy of the encoded variable, in order to make the values obtained with the different kinematic features comparable. For example, the Time variable contains 82 bins, so in principle, a perfectly informative cell could transmit $\log_{2}\left(82\right)\approx 6.3$ bits. The variable Direction, instead, contains only 2 bins, so the most informative cell could transmit at most 1 bit. When information values are normalized, the Time feature is still the most informative one, implying that its prevalence is not a consequence of the large number of bins it contains. The code indeed contains information about features that are not described by the other variables. The other features, however, appear flatter in panel C than in panel B. In particular, the feature Direction encoded negligible raw information values (panel B), but those values are a non-negligible amount of the maximal attainable values. Hence, the only reason the information about Direction is low, is that it contains only two bits, not because selectivity is poor.

To assess the significance of the differences in the values obtained for the two brain areas, the three neural codes, and the six kinematic features, we ran statistical tests for all 64 × 64 combinations of area + code + feature. The detailed procedure and the results are presented in Appendix A.

When the analysis of Figure 4 was repeated with all kinematic features, the results obtained with the variable SignedPosition were similar to those of Time. The mutual information of all other kinematic features were monotonically decreasing functions of the mean firing rate.

When the analysis of Figure 5A,B was repeated using kinematic features different from Time, similar results were obtained in both brain areas, with information and correlation values that either remain unchanged, or decrease slightly. Except for the case of Position and SignedPosition in H, for which both the information and the correlation between the firing and the theta code increased, as compared with Time ($I^{b}=15\%$ and 19%, and $c^{b}=0.34$ and 0.43, for Position and SignedPosition, respectively). A visual inspection of the firing properties of the neurons that employ both codes reveals place cells that precess in theta.

When the analysis Figure 5A,B was repeated using other kinematic features, the result was approximately the same as with Time, with slightly smaller values for the correlation between the information of the rate code and any of the phase codes, but slightly larger for the correlation between the information values of the two phase codes.

We next checked whether positions and velocities were better encoded in their signed or unsigned versions by each neural code. This point was addressed by calculating the amount of synergy $S_{k}$ between position and direction, and also between velocity and direction (see Equation (3) in Section 4.6). If the information about a signed variable was significantly higher than the sum of the information of the unsigned variable and the information about direction, the neural code represented conjunctions of kinematic features. In these cases, $S_{k}>0$. The opposite case appeared when $S_{k}<0$, resulting in redundant encoding.

**Figure 7:**
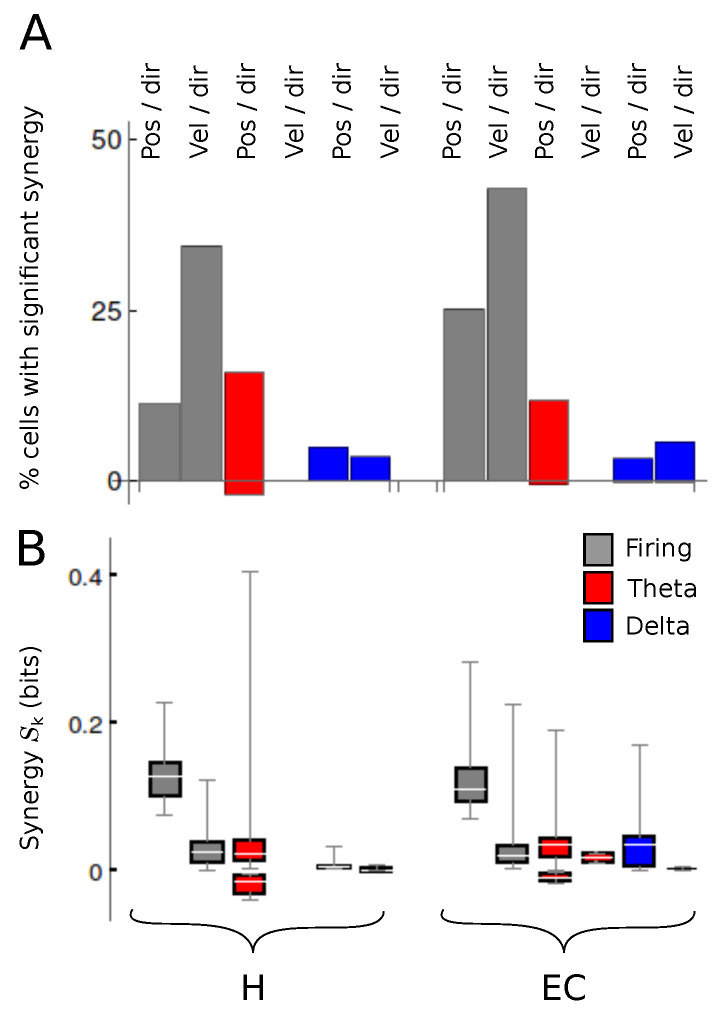
**Amount of synergy and redundancy between pairs of kinematic features**. (**A**) Percentage of recorded cells for which the encoding of position and direction, or of velocity and direction, is synergistic, in different brain areas or neural codes. Positive (negative) bars account for the cases where $S_{k}>0$ ($S_{k}<0$); (**B**) Box histograms displaying the amount of synergy between the two kinematic attributes, with positive and negative values displayed separately. Only significant values are included. Parameters of box histograms: same as in Figure 3.

In Figure 7 we see that the firing rate code represents position and direction synergistically in both areas. The same is true for velocity and direction. With the firing rate code, $S_{k}$ is never negative, implying that direction and position (or direction and velocity) are never encoded redundantly. From Equation (4) we see that redundancy is only possible if the two tested stimulus attributes are statistically related, that is, if I(Position;Direction)>0, or if I(Velocity;Direction)>0. These two mutual informations involve only kinematic variables, and are solely determined by the experimental paradigm. In the task analyzed here, I(Position;Direction)=0 and I(Velocity;Direction)=0, since for each position and each velocity, there are two possible directions of motion, both with equal probability. For this reason, this experiment rules out the possibility of finding redundancy. Some other pairs of attributes, as for example position and velocity, are correlated by the behavioral task. Since such correlation is determined by the experiment, and not by the neural code, the analysis of synergy between attributes is here restricted to position and direction, and to velocity and direction.

The theta code, instead, shows a few examples of redundancy between direction and position. These cases appear because in the phase codes, not all time bins are used to calculate the mutual information. The firing phase of a neuron cannot be calculated if the cell remains silent, so whenever a mutual information is calculated, the silent time bins are discarded from the set of stimuli. If the number of discarded bins occurring during the right run differs from those during the left run, a correlation between position and direction is likely to appear. Redundancy in a phase code, hence, can only be observed in neurons with unequal firing rates in the two running directions. In addition to these few redundancy examples, there is a large number of cells that encode position and direction synergistically. The theta phase, hence, is modulated by conjunctions of location and direction, and not by each attribute separately. Quite notably, no cells were found (neither in H nor in EC) with significant amounts of synergy or redundancy between velocity and running direction. A few neurons in both brain areas encoded position and direction, as well as velocity and direction, synergistically with the delta code. No instances of redundancy were found in the delta code.

### 2.5. The Encoding of Kinematic Variables by Pairs of Neurons

Finally, we verified whether pairs of neurons that were recorded simultaneously encoded information synergistically, redundantly or independently. Specifically, we determined whether the information encoded by pairs of neurons was equal (independent), larger (synergistic) or smaller (redundant) than the sum of the informations encoded by each member of the pair [25,26,27]. This analysis could only be performed in the rate code, since the phase codes are undefined in time bins where no spikes are fired, and those bins varied from neuron to neuron. The rate code, instead, is defined for all temporal bins. We modeled the conditional probability distribution that two neurons respond with firing rates $r_{1}$ and $r_{2}$ with a bivariate Gaussian probability distribution $P(r_{1},r_{2}|\mathrm{time})$ characterized by two mean values, two variances and a correlation coefficient, all parameters estimated from the experimental data (see Methods, Section 4.5 and Section 4.6).

**Figure 8:**
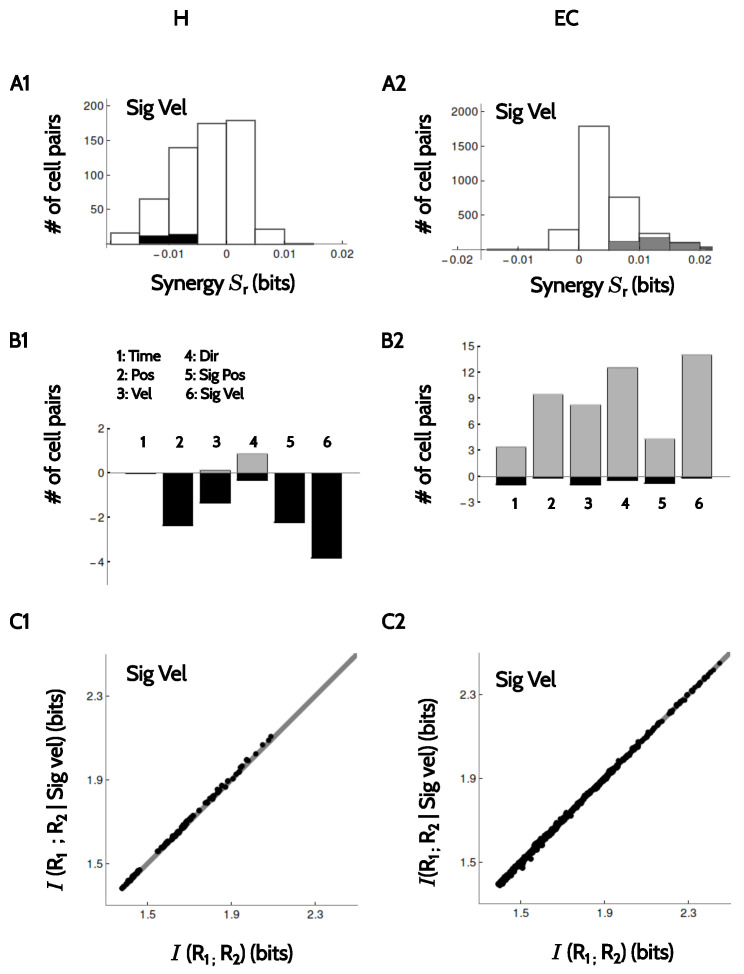
**Amount of synergy and redundancy between pairs of neurons**. (**A1**,**A2**) Histograms with the amount of synergy $S_{r}$ defined in Equation (5) in pairs of simultaneously recorded neurons in H and EC for signed velocity with the firing rate code. Significant values are marked in gray and black, depending on their sign; (**B1**,**B2**) Percentages of pairs of cells encoding significant amounts of synergy in the 6 tested kinematic attributes. Black and gray: redundant and synergistic encoding, respectively; (**C1**,**C2**) Scatter plot of ${\langle I\left(R_{1};R_{2}|S\right)\rangle }_{S}$ vs $I(R_{1};R_{2})$ for S=sigvel. Each data point is a pair of neurons. The amount of synergy $S_{r}$ is equal to the displacement from the diagonal.

In Figure 8(A1,A2), we see the histogram of the obtained synergy values for H and EC, respectively, for the example of signed velocity. Similar behavior was observed with other kinematic features. Notably, most of the values were not significant. In H a few pairs were significantly redundant, and in EC, a few were significantly synergistic. In B1–B2, we show the percentages of pairs in each area with significant amount of synergy (positive values) or redundancy (negative values) for different kinematic attributes. In H (Figure 8(B1)), pairs were always redundant, except for the encoding of running direction. In EC (Figure 8(B2)), both redundant and synergistic pairs were found, with a preference for synergy. However, in both areas the fraction of cells with significant amounts of synergy (positive or negative) was small. In addition, for all stimulus attributes, the actual values of the synergy were low when compared to the mutual information encoded by the members of each pair. Indeed, Schneidman et al. [27] showed that if $R_{1}$ and $R_{2}$ are the firing rates of the two cells in the pair and *S* is a given kinematic feature, then $S_{r}={\langle I\left(R_{1};R_{2}|S\right)\rangle }_{S}-I\left(R_{1};R_{2}\right)$. Hence, $S_{r}$ in a given pair is equal to the distance from the diagonal of the data point representing that pair in Figure 8(C1,C2). The collection of points does not show a noticeable displacement from the diagonal, implying that even for the few pairs with significant synergy, the magnitude of such synergy is a small fraction of the information values.

## 3. Discussion

Here we explored the neural code with which populations of neurons in H and EC represent kinematic information. We tested three neural codes, one based on the firing rates of cells in 1-second windows, and two based on the phase-of-firing with respect to the theta and delta filtered LFP, respectively. Firing rate codes have been observed ubiquituously in the nervous systems, with or without fine temporal structure [5,14]. Phase codes, instead, are comparatively new, and so far, have only been reported in the theta [11,12] and delta [28,29] bands. Two theoretical studies [30,31], and an experimental one [32] have also identified a possible mechanism in which phase codes can be translated into a pattern-based firing code by bursting neurons.

The firing rate code recruited a larger number of cells and transmitted more information than the other two. The strategy to estimate information values with the rate code was different from the phase codes, since in one case we fitted the experimental data with Gaussian distributions, and in the other, with Von Mises distributions. Moreover, all time bins could be associated with a Gaussian distribution, but not with a Von Mises distribution, since the latter required the bin to contain a minimum number of spikes. Therefore, information estimates performed with the rate code include all temporal bins, but the phase codes typically involved a smaller number of bins. Due to these differences, we believe that comparisons between the information values of the rate and phase codes should be made with caution, since two different estimation methods are in play. Still, the rate code surpasses the phase codes both in number of cells and magnitude in a sufficiently large difference as to make its primacy tenable. Yet, just for precaution, we prefer not to underscore the exact numerical difference between the information encoded by the rate code and the phase codes, also in view of the findings in other studies [33].

The theta and delta codes, instead, were estimated with the same method, the only difference being that recordings were selected on the base of a high-quality LFP, a condition requiring a well-defined theta peak in the LFP, and not a delta peak [34]. The similarity between the two estimation methods, and the knowledge that at most the delta code was in disadvantage, allows us to conclude that the information encoded in the delta code is perhaps smaller, but by no means negligibly smaller, than the one in the well-known theta code. To our knowledge, this is the first study reporting a role of the delta phase in the encoding of kinematic information in the temporal lobe. From the examples and information values obtained in the two brain areas, we conclude that the delta code is mainly involved in representing the Time variable, singling out specific moments during the run, which cannot be straightforwardly associated with a position, a velocity, or a direction.

All three codes tended to be most informative in cells with low mean firing rates (Figure 4A–C). This property suggests that the principal cells are more involved than the interneurons in the encoding of kinematic information. We also found that the circular variance with respect to the theta-filtered LFP (Figure 4D–F) was not a good predictor of the amount of encoded information. The same result was obtained with the circular variance with respect to the delta-filtered LFP (data not shown). Therefore, we conclude that all three codes recruit cells with preferentially low firing rates, but with varying degree of locking to the theta or delta signals.

The rate code recruited approximately half the cells in both areas in the encoding of Time. The delta and theta codes involved approximately 25% and 15% of the cells, respectively. In the case of Position and DirectionalPosition, the percentages with the firing rate code reach as high as 60–80%. These values are larger than the ones usually reported with traditional studies, in which the encoding of kinematic variables is assessed with well-defined attributes as position, head direction or velocity. Moreover, the hippocampal encoding of space is usually associated with place cells, and the entorhinal with grid cells. The fraction of cells devoted to such paradigmatic codes, however, is fairly low, around 20% [7,24]. Yet, as remakable as regular-firing maps are, position-selective codes need not be restricted to them. At the very least, grid cells have been reported to vary in the degree of periodicity of the location of the fields [35] and the field-to-field peak firing rate [24,36]. The high percentages of cells encoding significant amounts of information about position found in this study, and also in other approaches with a similarly broad perspective [23,24], suggests a wider repertoire of coding strategies, that are not necessarily covered by the traditional categories.

If we assume that these percentages represent the probability to pick a cell that makes use of the rate, theta and delta codes respectively, Figure 5 provides evidence that, for any pair of codes, the probability that a cell employs the two codes is higher than the product of the two marginals. The normalization restrictions imply that the probability that a cell employs neither of the two codes is also higher than the one predicted for independent populations. We conclude that cells are not segregated into a rate population, a theta population and a delta population. They are rather segregated into informative or non informative clusters. This effect is most pronounced for conjunctive encoding with the theta and delta codes, for which also the information values are correlated. The correlation is less clear when comparing cells using the rate code, and one of the phase codes, except for phase-precessing neurons in H, encoding DirectionalPosition. In general terms, the informative population includes a subpopulation of phase neurons, in which delta and theta codes largely overlap. Cells seem to be apt or inapt to use a phase code, but do not seem to be highly selective to a single frequency band.

The firing rate code is the one in which conjunctions of attributes (as position + direction, or velocity + direction) is most synergistic. Moreover, the amount of synergy between attributes is comparable to the total amount of information encoded by signed variables. We therefore conclude that both in H and in EC, position and direction are mainly encoded in conjunction with running direction with the firing rate code. The same conclusion holds for the phase codes, although the information values are lower.

When assessing the amount of synergy encoded in the firing rates of pairs of neurons recorded simultaneously, small differences in the amount of redundancy and synergy were found in the two brain areas. However, the values were too small to be considered significant. We therefore conclude that pairs of cells tend to encode information independently.

## 4. Materials and Methods

### 4.1. Electrophysiology and Behavioral Task

**Figure 9:**
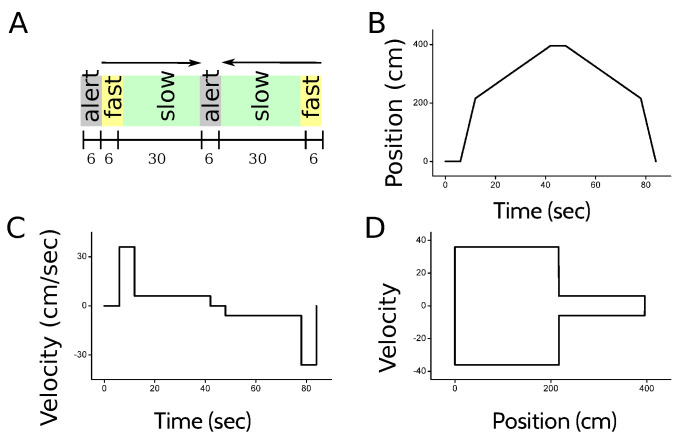
**Behavioral task**. (**A**) Each trial consisted of two episodes, the first running to the right, and the second to the left. Each episode began with an alert auditory signal lasting for 6 s, indicating the imminent start of the run. The animal was then compelled to run for 36 s at two different velocities, the order of which depended on the direction of motion; (**B**,**C**) Position and velocity of the cart as a function of time. The resting interval between the go and return journeys is not represented in the graph, because its duration was variable. During this interval, the animal took the reward, and was turned around in the cart; (**D**) Velocity as a function of position.

The activity of individual cells, as well as the LFP, was recorded in repeated trials in the hippocampus (CA1 and CA3) and the entorhinal cortex (layers II and III). The experiment was designed to ensure that in all trials, the trajectory of the animal was the same, in order to gather enough statistics in each position, velocity and direction of motion. The animal was placed inside a bottomless cart that moved along a 4 m long linear track. The position, velocity and direction of motion of the cart was controlled by a computer [7]. Since the cart had no floor, the animal had to engage in active locomotion at the experimenter-determined speed in order to reach the end of the track, where a food reward was delivered. At the beginning of each trial, the animal was placed inside the cart. A 6-s beep of increasing pitch indicated the beginning of the next run. After the beep, the cart started moving to the right at a speed of 36 cm/s. Halfway along the track, the cart slowed down to 6 cm/s until it reached the end of the track, where the cart stopped and the rat was rewarded. The animal then turned around to face the opposite end of the cart. The return run was also announced with a 6-sec beep, after which the cart started moving at 6 cm/s, for 30 s. Then, the cart was accelerated to 36 cm/s until it reached the initial position of the track. Figure 9 illustrates the behavioral task.

Typically, a session consisted of 10–25 runs on the linear track, all of them lasting at most 36 min.

### 4.2. Representation of Kinematic Variables

**Figure 10:**
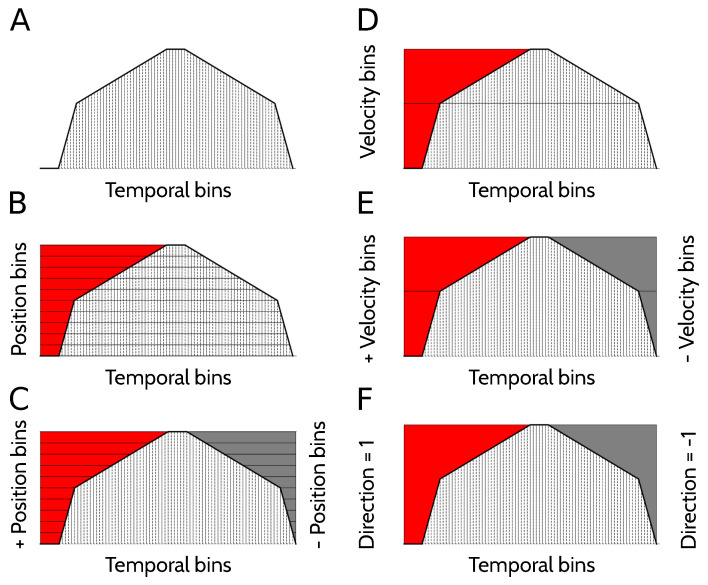
**Binning of kinematic variables**. (**A**) Temporal bins; (**B**) Position bins on the vertical axis, as a function of time; (**C**) Signed position bins (vertical axes on the left and right) as a function of time; (**D**) Direction of motion bins, on the left and right vertical axes. There is an additional bin for the alert periods where the rat has velocity zero; (**E**) Velocity bins, on the left vertical axis. There is also a zero-velocity bin; (**F**) Signed velocity bins, on the left and right vertical axes. There is also a zero velocity bin.

To estimate the amount of information that different neural codes carry about position, velocity, direction of motion, or combinations of these variables, a suitable representation of the kinematic state of the animal is needed. Each trial was divided in 82 temporal bins (Figure 10A), each lasting for 1 s. In this paper, the specification of the temporal bin along the trial defines the variable *Time* (Figure 10A), and provides the most complete description of the kinematic state of the animal, since the position, velocity and direction of motion are all functions of time.

The variable *Position* specifies the spatial location of the animal (Figure 10B), and is divided into 12 bins. Two of them correspond to the location of the cart at the beginning and the end of the track while the alarm is ringing. Another five locations bins are assigned to the state of fast running, each of them labeling the location of the animal in one of the 5 s in which the cart moves at 36 cm/s. There are five more position bins during slow running, each associated to the location of the cart during 6 consecutive seconds in which the cart moves at 6 cm/s. Position bins during the running state have all equal length, but correspond to time intervals of different durations. Since the running velocity varies throughout the trial, position bins during slow running are associated to time intervals 6 times longer than those of fast running. The marginal probability of the slow-running bins, hence, is 6 times larger than that of the fast-running bins.

We also worked with a kinematic variable defined as *Signed position* (Figure 10C), with positive positions corresponding to the locations in which the animal is running to the right (or preparing to run to the right), and negative positions for left-directed runs. There are 22 bins of signed position. The variable *Direction of motion* has 2 bins: left, right (Figure 10D). When the animal is waiting at the beginning of the track for the run to start, the direction is the one of the imminent run, that is, the one towards the head is pointing to. The feature *Velocity* has 3 bins: fast, slow and still (Figure 10E). *Signed velocity* has 6 bins: still right, fast right, slow right, still left, fast left and slow left. (Figure 10F).

### 4.3. Amount of Spectral Power in Different Frequency Bands of the LFP

The LFP was filtered in the theta (6–12 Hz) and delta (1.5–4 Hz) bands. Filtering consisted of (a) applying a fast Fourier transform to the LFP, (b) assigning amplitude 0 to bins with frequency outside the desired band and (c) transforming the result back to the temporal domain. Since we often also needed the phase of the signal, the bandpass filter was sometimes followed by the Hilbert filter (assigning 0 amplitude to negative frequencies in the Fourier domain) to obtain the discrete-time analytic signal as a result. The exact form of the hilbert filter can be found in the MATLAB function hilbert(). The output of the Hilbert filter was used as an argument to the MATLAB function angle(), with which the phase was calculated.

**Figure 11:**
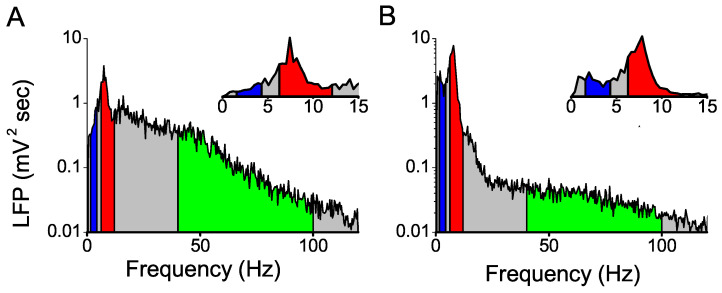
**Average spectral power density of the LFPs**. (**A**) Hippocampus; (**B**) Entorhinal cortex. Logarithmic scales in the *y*-axis of the main figures, and linear scale in the amplified view of the insets. Delta band: 1.5–4 Hz. Theta band: 6–12 Hz. Gamma band: 60–100 Hz.

Phase codes are only possible for frequency bands with significant power in the LFP. To identify the frequency bands with non-negligible power, in Figure 11 we show the spectral power density in both the hippocampus and the entorhinal cortex.

In both areas, the spectrum contained a clear maximum in the theta band (Figure 11). An additional and less prominent peak was observed in the delta band of the LFP recorded in the entorhinal cortex, comprising between one third and one half of the total power in the theta band. In the hippocampus, slow oscillations did not give rise to a low-frequency peak, and yet, the power in the delta band was far from negligible, comprising about one third of the power in the theta band. The first harmonic of the theta frequency gave rise to an additional local maximum at about 16 Hz, after which the power spectrum decayed monotonically.

### 4.4. Circular Statistics

The phase of the LFP is a circular variable, here defined between −π and π. The mean 〈ϕ〉 and the variance $\sigma_{\phi }^{2}$ of a collection of angles $\alpha_{i}$ with 1≤i≤n is

(1) $$\begin{array}{ccc} \langle \phi \rangle & = & \mathrm{Arc}\;\mathrm{Tan}(S/C) \end{array}$$

(2) $$\begin{array}{ccc} \sigma_{\phi }^{2} & = & 1-\sqrt{S^{2}+C^{2}}, \end{array}$$

where *S* and *C* are the average sine and cosine values, respectively [37]

$$\begin{array}{ccc} S & = & \frac{1}{n}\;\sum_{i=1}^{n}\sin\alpha_{i},\\ C & = & \frac{1}{n}\;\sum_{i=1}^{n}\cos\alpha_{i}. \end{array}$$

The circular variance is always between 0 and 1. When all angles $\alpha_{i}$ coincide, the circular variance vanishes. When they are evenly distributed on the circle, the circular variance reaches its maximal value. A small number of samples, however, need not represent faithfully the statistics of the underlying distribution. For example, when a few angles are sampled from a uniform probability distribution, it is highly unlikely to obtain values that are evenly distributed in the interval (−π,π). So even if the circular variance associated to the uniform distribution is unity, the estimated value is typically lower. The circular variance is in fact negatively biased: On average the value obtained for a finite set of samples is smaller than the value of the underlying distribution. It is, therefore, important to determine whether the circular variance estimated from a few samples provides enough evidence to conclude that the underlying distribution is uneven or, on the contrary, whether that same value could have arisen from a uniform distribution. We therefore worked with a null hypothesis, under which the underlying distribution is uniform. To assess how likely it would be to obtain the measured circular variance under this hypothesis, we compared the measured value with the distribution of values that are expected from the null hypothesis when sampled the same number of times as the real data. If the real circular variance is smaller than 99% of the values obtained from a uniform distribution, the measured value is considered significant, and the underlying distribution, non-uniform.

### 4.5. Estimation of Encoded Information

The amount of information that a given physiological variable *Y* (as firing rate, or phase-of-firing with respect to the filtered LFP) and a kinematic variable *X* (as Time, Position, Direction, etc.) was computed by Shannon’s mutual information [38,39]

$$I\left(X;Y\right)=\sum_{x}P\left(x\right)\int P\left(y|x\right)\;\log_{2}\left[\frac{P(y|x)}{P(y)}\right]\;dy,$$

where P(x) is proportional to the amount of time that the animal spent with kinematic variable X=x. In other words, if each trial has duration $T_{\mathrm{trial}}$, and if in each trial the animal spends an amount of time $T_{x}$ with X=x, then $P\left(x\right)=T_{x}/T_{\mathrm{trial}}$. In turn, P(y|x) is the probability that the physiological variable *Y* take the value *y* conditional on X=x.

**Figure 12:**
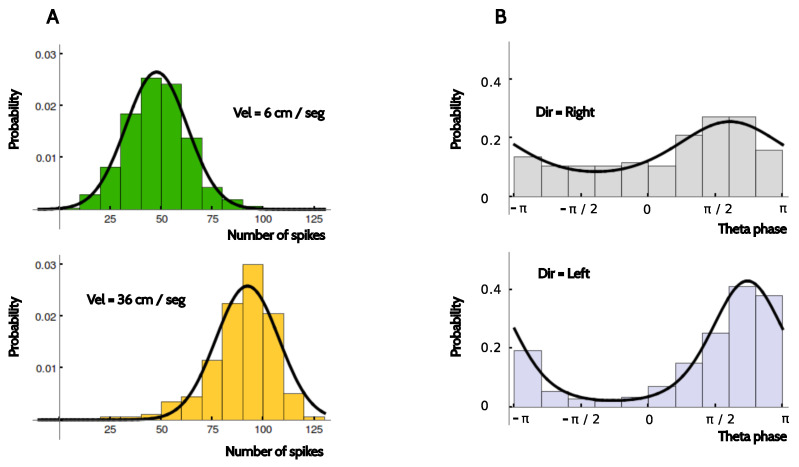
**Modeling the conditional response distributions**. Comparison between the histogram of the measured response variables and the corresponding fits, for two example neurons and codes. (**A**) Firing rates obtained from an entorhinal neuron for two different running speeds. Note the different in scale of the *x* axes. The two velocities induce markedly different mean firing rates; (**B**) Phase of firing with respect to theta of an entorhinal neuron for the two different running directions. The two directions give rise to distributions of markedly different widths.

The number of trials with the electrodes in the same position was around 15. Without additional assumptions, this small number of samples rules out the possibility of making a reliable estimation of the distributions P(y|x). We therefore assumed that in the firing rate code, the distribution P(rate|time) could be approximated by a Gaussian distribution with the experimentally measured mean and variance. In the two phase codes, P(ϕ|time) was modeled as a Von Mises distribution with the mean and concentration values extracted from the data. These assumptions were based on the observation that the conditional probabilities were unimodal, and that the data were typically sufficient to make reasonable estimations of the mean value and the width of the distributions, as shown in the examples of Figure 12.

We checked that in the case of the firing rate code, using a Poisson fit (instead of a Gaussian fit) produced only a minor variation of the obtained information values. The Gaussian model had the advantage to be easily extensible to the bivariate case, when evaluating the amount of information encoded by pairs of simultaneously recorded neurons. In this case, the distribution $P(r_{1},r_{2}|\mathrm{time})$ was modeled as 2-dimensional Gaussian, which only requires the additional estimation of the correlation coefficient between the rates $r_{1}$ and $r_{2}$ of the two neurons, for each temporal bin. The parametrization of response probability distributions conditional to a given stimulus with specific models has been widely employed before in the framework of Fisher-information-based measures (for example, ref. [40,41] used a multivariate Gaussian model), and sometimes also for the estimation of Shannon-based measures (for example, ref. [4] employed a binary model, whereas [42] used a Gaussian model.)

The Gaussian fit of the probability P(r|Time=t) of firing at rate *r* conditional on the temporal bin Time=t was calculated by estimating the mean spiking rate and the variance at each time *t*. If the number of spikes in that bin was smaller than 10 (considering all trials) or the number of trials containing spikes in that bin was smaller than 5, the variance was taken as the minimum value of the variances corresponding to Time≠t. This choice ensured conservative information estimates, since variances obtained with only a few samples are unreliable.

To calculate the probability P(r|S=s) of firing *r* spikes conditional on the kinematic feature *S* taking the value *s*, we marginalized over all the Gaussian fits of the distributions P(r|Time=t) at time bins *t* compatible with the condition S=s. Hence, the distribution P(r|S=s) was typically not Gaussian. With this definition of P(r|S=s), we ensured the validity of the data processing inequality: Since given the value of Time, the value of *S* is determined, I(R;Time)≥I(R;S).

When estimating the information encoded by the firing rates of pairs of neurons recorded simultaneously, the conditional probability $P(r_{1},r_{2}|\mathrm{time})$ was modeled as a bivariate Gaussian distribution characterized by the means $\langle r_{1}\rangle$ and $\langle r_{2}\rangle$, variances $\sigma_{1}^{2}$ and $\sigma_{2}^{2}$ and correlation coefficient ρ. To ensure a reliable estimate of ρ, if the number of spikes in that bin was smaller than 20 (considering all trials) or the number of trials containing spikes in that bin was smaller than 5 (of any of the two neurons), we set ρ=0. By marginalizing $P(r_{1},r_{2}|\mathrm{time})$ in the value of “Time” and keeping $r_{1}$ and $r_{2}$ fixed, we obtained the distributions $P(r_{1},r_{2})$ that were used to estimate the information $I(R_{1};R_{2})$ between the rates.

The von Mises fit of the probability P(ϕ|Time=t) of firing at phase ϕ conditional on the temporal bin Time=t was calculated by estimating the circular mean and the circular variance of all spiking phases obtained at that particular time bin. If fewer than 10 spikes existed, or if less than 5 trials contained spikes, the bin Time=t was discarded from the list of values that was taken by kinematic variable Time in the estimation of information. Just as with the firing rate, to calculate the probability P(ϕ|S=s) of firing at phase ϕ conditional on the kinematic feature *S* taking the value *s*, we marginalized over all the von Mises fits of the distributions P(ϕ|Time=t) at time bins *t* compatible with the condition S=s. Hence, the distribution P(ϕ|S=s) was typically not von Mises. The marginal distributions p(r) and p(ϕ) were also obtained by marginalizing the conditional distributions p(r|t) and p(ϕ|t), respectively.

### 4.6. Estimation of the Amount of Synergy

When either the kinematic or the neuronal variable (or both) are defined in terms of two components, one may wonder whether the information encoded by the pair of components is equal, more, or less than the sum of the informations encoded by each component separately. For example, the variable “signed velocity” can be interpreted as a 2-dimensional vector (Direction,Velocity), where the component Direction can take the values {1,−1}, depending on whether the head of the animal points to the right or to the left, and the component Velocity can be 0, 6 or 36 cm/s, depending on the running speed. If the response variable *Y* represents the conjunction of direction and velocity, but not each variable separately, I(Signedvel;Y) is expected to surpass I(Direction;Y)+I(Velocity;Y). In this case, direction and velocity are encoded synergistically. If, instead, the two aspects are represented independently, I(Signedvel;Y) should be approximately equal to I(Direction;Y)+I(Velocity;Y). Finally, if the encoding of the running speed could be used to infer the direction, I(Signedvel;Y) should be smaller than I(Direction;Y)+I(Velocity;Y), and the code is redundant.

The amount of synergy between two kinematic variables $X_{1}$ and $X_{2}$ mediated by the response variable *Y* is defined as

(3)Sk=I(X1,X2;Y)−I(X1;Y)−I(X2;Y),(4)=〈I(X1;X2|Y)〉Y−I(X1;X2),

where the subscript in $S_{k}$ stands for “synergy in the **k**inematic variables”, and the equality between the two expressions was demonstrated by Brenner et al. [26]. Analogously, when considering the responses of two neurons recorded simultaneously with activities $Y_{1}$ and $Y_{2}$, the synergy observed in the encoding of kinematic variable *X* is

(5)Sr=I(X;Y1,Y2)−I(X;Y1)−I(X;Y2),(6)=〈I(Y1;Y2|X)〉X−I(Y1;Y2),

where the subscript in $S_{r}$ stands for “synergy in the **r**esponse variables”.

### 4.7. Statistical Significance of Information Measures

Estimates of mutual informations obtained with a limited number of samples are positively biased [43,44,45,46]. To assess whether each obtained value was significant, we shuffled all spikes in each trial, mimicking the situation in which P(r|s)=P(r) and P(ϕ|s)=P(ϕ). Information values were calculated for 100 independent realizations of the shuffling. The information value obtained in the real experiment was considered significant if it was larger than all, or at most a single, of the shuffled values. The same criterion was used to assess the significance of the information encoded by pairs of neurons, based on the bivariate distributions $P(r_{1},r_{2}|s)$.

For each recorded area, neural code, and kinematic feature, we determined the fraction of cells encoding significant amounts of information. To assess whether the fractions obtained in two different conditions were significantly different or not, we evaluated the null hypothesis that they were equal, and estimated the probability to obtain two fractions that differed in at least as much as the difference observed in the experimental data, just because the number of cells is limited. Fractions of cells were considered significantly different if the experimental difference was larger than 99% of the trials evaluated under the null hypothesis. In the case of the information between rates $I(R_{1};R_{2})$, we considered significant the experimental values that surpassed 50 shuffled trials.

To assess whether an estimated synergy is significant, we shuffled the response vectors $\left(y_{1},y_{2}\right)$ obtained in different temporal bins 50 times, mimicking the situation in which the conditional probability $P(y_{1},y_{2}|\mathrm{time})$ was drawn from the marginal probability $P(r_{1},r_{2})$. A synergy value was considered significant if the number obtained with the real data was larger than all but one of the values derived from the 100 shuffled instances.

### 4.8. Assessing the Degree of Joint Encoding with Different Neural Codes

**Figure 13:**
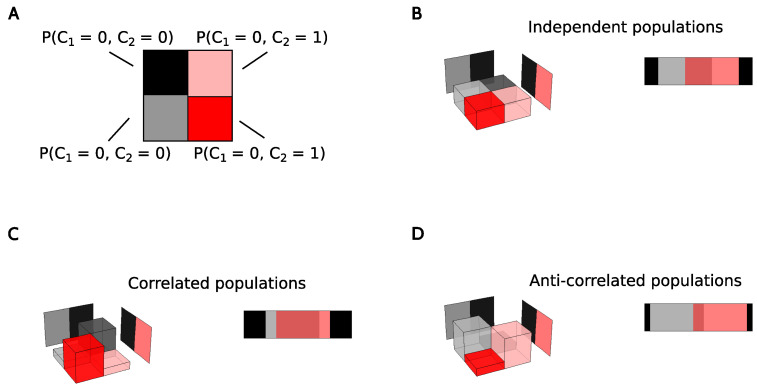
**Idealized examples of pairs of codes with varying overlap throughout the population of neurons**. (**A**) Color convention used in panels (**B**–**D**); (**B**) Joint probability distributions (blocks) and the corresponding marginal (planes) of an example case with two codes that are independent. The rectangular stripe shows that the fraction of neurons employing both codes is the one expected from the total number of neurons; (**C**) Codes with a tendency to coexist in the same neurons; (**D**) Codes with a tendency to be segregated into different cells.

Each cell did or did not encode a significant amount of information about a specific kinematic feature with the rate, the theta and the delta codes. To verify whether codes tended to co-occur in the same cell or, on the contrary, tended to be segregated in different neurons, for each cell we defined three binary variables $C_{r},C_{\theta },C_{\delta }$ that took the values 1 or 0, depending on whether the cell did or did not transmit a significant amount of information about the variable Time in the rate code, the theta code and the delta code, respectively. We assumed that each area could be characterized by a joint probability distribution $P(c_{r},c_{\theta },c_{\delta })$, and that the collection of cells recorded in each area constituted a sample of the corresponding distribution. Marginalizing over one of the three codes, we obtain bivariate distributions $P\left(c_{r},c_{\theta }\right),P\left(c_{\theta },c_{\delta }\right)$ and $P(c_{\delta },c_{r})$. In Figure 13, we illustrate the distributions obtained for an idealized case in which there are two neural codes, each of which recruits half of the neurons in the populations. Panel B represents the case in which the two codes are independent of one another, panel C when they tend to coexist in the same cells, and panel D when they tend to be segregated in different neurons.

If the population of cells employing one neural code is independent from the population using another code, then the knowledge that a given cell has $C_{i}=1$ cannot be used to predict the value of $C_{j}$, and vice versa. This is shown graphically in Figure 13B, where $P\left(c_{i},c_{j}\right)=P\left(c_{i}\right)P\left(c_{j}\right)$. Mathematically, this means that the mutual information $I^{b}\left(C_{i};C_{j}\right)$ between $C_{i}$ and $C_{j}$ vanishes, with

(7) $$I^{b}\left(C_{i};C_{j}\right)=\sum_{ab}P_{ij}\left(ab\right)\;\log_{2}\left[\frac{P_{ij}\left(ab\right)}{P_{i}\left(a\right)\;P_{j}\left(b\right)}\right],$$

where the sum runs over all pairs ab∈{00,01,10,11}, and the supra-script in $I^{b}$ stands for “binary”. A vanishing information implies that the collection of cells that encodes information with one of the tested codes is independent from the other. Positive information values may either indicate that the co-occurrence of two codes in one same cell is more frequent than random (Figure 13C), or alternatively, that if one code is used, then the other is not (Figure 13D). To discriminate between these two situations, we calculate pairwise linear correlations between the variables $C_{r}$, $C_{\theta }$ and $C_{\delta }$. Positive values of the Pearson correlation coefficient implies that codes tend to co-occur in the same cell, whereas negative values are found when cells using each code tend to be segregated. The Pearson correlation between the set of neurons encoding information with codes *i* and *j* is defined as

(8) $$\begin{array}{ccc} c_{ij}^{b} & = & \frac{\langle C_{i}C_{j}\rangle -\langle C_{i}\rangle \;\langle C_{j}\rangle }{\sqrt{\left(\langle C_{i}^{2}\rangle -{\langle C_{i}\rangle }^{2}\right)\;\left(\langle C_{j}^{2}\rangle -{\langle C_{j}\rangle }^{2}\right)}}\;\\ & = & \frac{P_{ij}\left(11\right)-P_{i}\left(1\right)P_{j}\left(1\right)}{\sqrt{P_{i}\left(1\right)\left[1-P_{i}\left(1\right)\right]\;P_{j}\left(1\right)\left[1-P_{j}\left(1\right)\right]}}\;\\ & = & \frac{P_{ij}\left(00\right)-P_{i}\left(0\right)P_{j}\left(0\right)}{\sqrt{P_{i}\left(1\right)\left[1-P_{i}\left(1\right)\right]\;P_{j}\left(1\right)\left[1-P_{j}\left(1\right)\right]}} \end{array}$$

where the supra-script b stands for “binary”.

For each pair of physiological variables (i,j)—that is, for the pair (i,j)=(r,θ), the pair (i,j)=(θ,δ) and the pair (i,j)=(δ,r)—we selected the cells that encoded significant amounts of information about the variable Time in both physiological variables, and with these cells, we calculated the Pearson correlation coefficient between I(Time;i) and I(Time;j). The result is displayed in Figure 5C. For each pair of codes i/j, we also performed a linear fit I(Time;j)=αI(Time;i)+β. In Figure 5D we report the slopes α of the fits obtained with different physiological variables (i/j) and in the two brain areas.

### 4.9. Data Selection

Information values were only calculated for sessions where the LFP contained a well-defined peak in the theta band. The peak value of the spectral power density in the theta band was required to be at least twice as larger than the density at any of the inflection points at either side of the peak. This condition was fulfilled by 220 trials (out of 649) in H, and 1186 (out of 1249) in EC.

## 5. Conclusions

The study of the neural code aims at revealing the dictionary with which external stimuli are translated into neural responses [5,14]. This endeavour implies the identification of the stimulus and response features that are encoded, and the probabilistic mapping between them. One research line explores the dictionary in terms of the adequacy with which the original stimuli may be recovered after a decoding procedure [30,47,48,49,50], whereas another line focuses only on the encoding process [27]. In the end, the evolutionary advantage of perceptual systems is to produce adequate behavior, which may or may not require the original stimulus to be decoded. Here we studied the neural code of hippocampal and entorhinal cells of an awake and behaving rat in the framework of encoding. We demonstrated that the use of a broad repertoire of candidate input and response features allowed us to identify novel encoding strategies. In particular, the phase-of-firing with respect to the delta-filtered LFP was shown to encode kinematic information. To a large extent, this information refers to specific time points along the trajectory, that cannot be easily associated with a single position, velocity, or direction of motion, but rather, a conjunction of several features. We interpret these results as a proof of concept, that should be further confirmed with more varied experimental protocols, and a larger number of experimental animals.

## Figures and Tables

**Figure A1:**
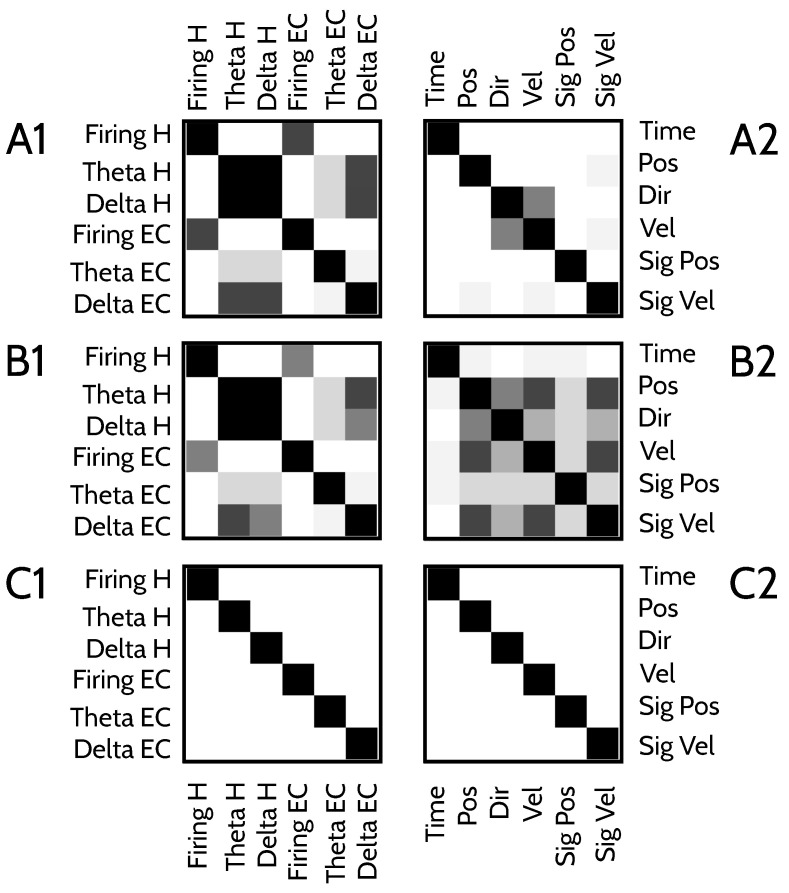
**Significance between the different values reported in Figure 3 and Figure 6**. Left panels: Grey-scale representation of the significance matrices (see Methods) obtained for each pair of neural code and brain area, averaged across the 6 different kinematic features. Right panels: Gray-scale representation of the significance matrices obtained for each pair of kinematic feature, averaged across the 6 different conditions (Firing H, Theta H, Delta H, Firing EC, Theta EC, Delta EC). (**A**) Significance between the collections of information values expressed in bits, as in Figure 3B and Figure 6B; (**B**) Significance between the collections of information values expressed as a fraction of the maximal information, as in Figure 6C; (**C**) Significance between the fractions of cells that encode significant amounts of information, as in Figure 3A and Figure 6A.

## Abbreviations

H	Hippocampus
EC	Entorhinal cortex
LFP	Local field potential

## Appendix A. Significance Analysis

Figure 3 and Figure 6 display the number of cells encoding significant amounts of information (panel A), and also the histograms of information values (panels B and C). To assess whether two percentages of cells were significant, we ran the test described in Section 4.5. To determine whether the mean information values corresponding to two different histograms were significantly different or not, we performed a Student *t*-test with a significance threshold of p=0.01. For each comparison, we defined a significance variable η that could be either 0 (not significantly different) or 1 (significantly different). There are 6 kinematic variables (Time, Position, Velocity, Direction, Signed Position and Signed Velocity), 3 neural codes (rate, theta and delta) and 2 brain areas (H and EC), so in total, there are 36 percentages of informative cells, 36 information values, and 36 normalized information values. All comparisons define 36×36η values for each quantity.

Figure A1 displays the obtained η values averaged across kinematic features (left column) or code and area (right column). The diagonal is always black, because all tests result in η=0 when the comparison involves two equal conditions. In A1 and B1 we note that the mean information values obtained with the theta and delta codes are not significantly different in the hippocampus (black boxes). Moreover, the mean information of the phase codes in H is rarily significantly different from the phase codes in EC (gray squares). In addition, the mean information values in H is also similar to the values in EC (gray). In A2 we see that direction and velocity (the two features with smallest information) have similar mean information values, all other kinematic features with more distinguishable means. When information values are normalized with their maximal attainable value (the entropy of the stimulus set), however, most kinematic features have non-significantly different means, except for the attribute Time, and (to a lesser extent), SignedPosition (panel B2). Panels C1 and C2 show that all pairs of fractions of cells encoding significant amounts of information are always significantly different.

## Appendix B. Tables

**Table A1:** Mean, standard deviation and maximal information values of Figure 3B.

Region	Code	N Cells	Mean (bits)	St. Dev. (bits)	Maximum (bits)
H	firing	107	0.45	0.20	0.96
H	theta	63	0.13	0.066	0.37
H	delta	38	0.098	0.11	0.74
EC	firing	754	0.44	0.22	1.4
EC	theta	297	0.11	0.064	0.43
EC	delta	188	0.089	0.038	0.35

**Table A2:** Mean, standard deviation and maximal information values of Figure 6B.

Region	Code	Feature	N Cells	Mean (%)	St. Dev. (%)	Maximum (%)
H	firing	Time	107	8.22	3.87	17.31
H	firing	Position	148	4.65	3.61	18.52
H	firing	Direction	72	5.05	6.47	33.42
H	firing	Velocity	116	5.34	3.73	18.41
H	firing	Signed Pos	115	6.36	3.48	16.85
H	firing	Signed Vel	147	5.00	3.81	21.30
H	theta	Time	62	3.96	4.80	36.62
H	theta	Position	47	0.77	1.18	7.58
H	theta	Direction	23	0.19	0.13	0.51
H	theta	Velocity	41	0.48	0.49	1.87
H	theta	Signed Pos	53	1.77	2.83	20.95
H	theta	Signed Vel	40	0.54	0.54	2.28
H	delta	Time	37	2.76	4.21	26.34
H	delta	Position	17	0.33	0.22	0.84
H	delta	Direction	19	0.13	0.09	0.30
H	delta	Velocity	16	0.36	0.23	0.93
H	delta	Signed Pos	20	0.58	0.52	2.15
H	delta	Signed Vel	22	0.31	0.26	1.09
EC	firing	Time	754	7.83	4.22	27.71
EC	firing	Position	870	3.74	2.92	19.37
EC	firing	Direction	601	5.96	7.27	58.63
EC	firing	Velocity	742	4.42	4.82	46.30
EC	firing	Signed Pos	778	5.49	3.17	22.04
EC	firing	Signed Vel	949	5.14	4.92	34.07
EC	theta	Time	295	2.81	2.19	24.51
EC	theta	Position	274	0.41	0.47	3.51
EC	theta	Direction	216	0.36	0.70	6.29
EC	theta	Velocity	295	2.81	2.19	24.51
EC	theta	Signed Pos	261	1.61	1.19	7.58
EC	theta	Signed Vel	307	0.40	0.45	3.86
EC	delta	Time	188	2.26	2.05	25.70
EC	delta	Position	114	0.18	0.13	0.81
EC	delta	Direction	62	0.15	0.16	1.12
EC	delta	Velocity	104	0.18	0.13	0.89
EC	delta	Signed Pos	76	1.19	0.89	6.26
EC	delta	Signed Vel	130	0.18	0.12	0.80

**Table A3:** Mutual informations between the binary variables defined in Section 4.8 and shown in Figure 5A–C.

Region	Code *A*	Code *B*	I(A;B) in bits	I(A;B)/Min[H(A),H(B)]
H	firing	theta	0.032	3.7%
H	theta	delta	0.15	23 %
H	delta	firing	0.29	43 %
EC	firing	theta	0.042	5.2%
EC	theta	delta	0.11	18%
EC	delta	firing	0.25	39 %
